# ΔNp63 regulates Sfrp1 expression to direct salivary gland branching morphogenesis

**DOI:** 10.1371/journal.pone.0301082

**Published:** 2024-05-09

**Authors:** Theresa Wrynn, Sangwon Min, Erich Horeth, Jason Osinski, Satrajit Sinha, Rose-Anne Romano

**Affiliations:** 1 Department of Oral Biology, School of Dental Medicine, State University of New York at Buffalo, Buffalo, New York, United States of America; 2 Department of Stem Cell and Regenerative Biology, Faculty of Arts and Sciences, Harvard University, Cambridge, Massachusetts, United States of America; 3 Department of Biochemistry, School of Dental Medicine, State University of New York at Buffalo, Buffalo, New York, United States of America; University of California Davis, UNITED STATES

## Abstract

Branching morphogenesis is a complex process shared by many organs including the lungs, kidney, prostate, as well as several exocrine organs including the salivary, mammary and lacrimal glands. This critical developmental program ensures the expansion of an organ’s surface area thereby maximizing processes of cellular secretion or absorption. It is guided by reciprocal signaling from the epithelial and mesenchymal cells. While signaling pathways driving salivary gland branching morphogenesis have been relatively well-studied, our understanding of the underlying transcriptional regulatory mechanisms directing this program, is limited. Here, we performed *in vivo* and *ex vivo* studies of the embryonic mouse submandibular gland to determine the function of the transcription factor ΔNp63, in directing branching morphogenesis. Our studies show that loss of ΔNp63 results in alterations in the differentiation program of the ductal cells which is accompanied by a dramatic reduction in branching morphogenesis that is mediated by dysregulation of WNT signaling. We show that ΔNp63 modulates WNT signaling to promote branching morphogenesis by directly regulating *Sfrp1* expression. Collectively, our findings have revealed a novel role for ΔNp63 in the regulation of this critical process and offers a better understanding of the transcriptional networks involved in branching morphogenesis.

## Introduction

The salivary gland (SG) is an exocrine organ that produces saliva which plays an essential role in a number of important processes including lubrication, mastication, digestion, speech, taste and maintaining oral health [1–3]. In mice, submandibular salivary gland (SMG) morphogenesis commences at embryonic (E) day 11.5 (E11.5) with a thickening of the epithelium creating a placode. By E12.5, the thickened epithelium invaginates into the underlying mesenchyme forming an end bud connected to a stalk, that later serves as the main duct once the gland reaches maturation [4, 5]. As the gland continues to develop, it undergoes rapid proliferation and branching morphogenesis during which the end bud cells undergo clefting and branch elongation generating secondary ducts. As maturation progresses, successive rounds of end bud cleft formation, branching, and ductal elongation results in an intricate and complex ductal network [5]. Reorganization of the end buds and formation of the acini, the main secretory units of the salivary gland, also occurs. By E18.5, expansion of the acini and lumenization of both the ducts and acini nears completion, culminating in a continuous network of ducts connecting the acini to the oral cavity [5]. This also marks the establishment of the main epithelial cell types that comprise the SG, including the saliva producing acinar cells, the myoepithelial cells that surround the acini and aid in contractile extrusion of the saliva into the oral cavity via a well-developed ductal network comprised of ductal cells and supporting basal cells. Terminal differentiation of the saliva producing acinar cells and ducts, continues postnatally and by puberty, differentiation of the granular convoluted tubule is complete [4–7].

Wnt signaling is a conserved signaling pathway that plays important roles in various aspects of development including cell fate determination, cell migration and proliferation, tissue homeostasis and regeneration [8, 9]. Wnt signals are also critical for salivary gland morphogenesis where it is initially localized to the mesenchyme at E12 and by E13.5, it is expressed in the ductal cells where it is has been shown to play important roles in ductal differentiation. Indeed, during the early stages of salivary gland development, studies have revealed that inhibition of Wnt signaling is required for branching morphogenesis [10, 11]. Similarly, studies in the lung and lacrimal gland support these findings [12]. While several Wnt antagonists have been identified, a role for the secreted frizzled related proteins (sFRPs) in inhibiting Wnt activity has been reported in the salivary gland. More specifically, sFRP1 has been demonstrated to inhibit Wnt signaling and promote branching morphogenesis, in part through regulation of FGF signaling [10]. While a role for sFRP1 in branching morphogenesis is clear, the mechanisms regulating sFRP1 expression in the SG remain to be elucidated.

The transcription factor p63, specially the ΔNp63 isoform of p63, functions as a lineage-specific master transcription factor that is highly expressed in the basal and myoepithelial cells of epithelial-rich tissues and organs where it plays important roles in stem cell self-renewal, morphogenesis and directing differentiation programs [13–21]. During SG embryonic development, this transcriptional regulator is expressed in the epithelial cells of the developing salivary gland placode. As development proceeds, ΔNp63 is expressed in the initial stalk and the developing end buds, where it plays critical roles in directing SMG cell fate decisions as illustrated by the complete block in gland morphogenesis in animals with targeted deletion of this isoform [22–24]. Furthermore, lineage tracing studies have demonstrated that during SMG development, ΔNp63 expressing cells are multipotent and give rise to all the epithelial cell types of the gland [22]. While the critical importance of this transcription factor in SMG development has been well established, studies investigating the function of ΔNp63 in directing various developmental programs including branching morphogenesis have not been investigated, in large part due to the severe embryonic phenotype associated with loss of ΔNp63 in conventional knockout (KO) models.

To investigate the role of ΔNp63 during SMG development, we have generated a ΔNp63 inducible conditional knockout mouse model. We demonstrate that ablation of ΔNp63 results in alterations to the ductal cell differentiation program and reduced duct cell proliferation. Additionally, we find aberrant differentiation of the basal and myoepithelial cells which are accompanied by a dramatic loss to these stem/progenitor cell populations. Utilizing an *ex vivo* organ culture model system, we show that loss of ΔNp63 results in a striking defect in branching morphogenesis. To uncover the specific ΔNp63-driven regulatory networks through which this regulator functions to direct the branching morphogenesis program, we have utilized existing transcriptomic (scRNA-seq) and ChIP-seq data sets. Integrated analysis of these data sets reveals that ΔNp63 modulates Wnt signaling by directly regulating the expression of *sFRP1*, an antagonist of the Wnt signaling pathway, to direct branching morphogenesis. Taken together, our data uncovers a newly discovered role for the transcription factor ΔNp63 acting as an upstream regulator of the Wnt signaling pathway in directing salivary gland branching morphogenesis.

## Results

### Alterations to the ductal cell differentiation program in the absence of ΔNp63

**Fig 1:**
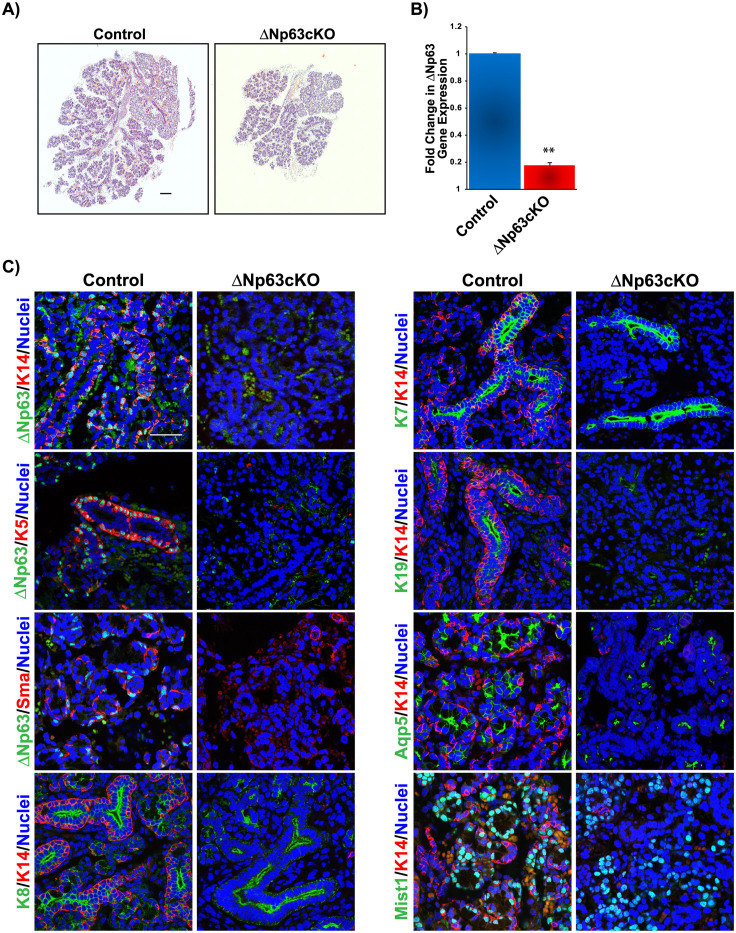
Histological and immunostaining analysis of submandibular salivary glands of mice with targeted deletion of ΔNp63. (**A**) H&E staining of E18.5 salivary glands from control and ΔNp63cKO mice. Compared to control, ΔNp63cKO glands are smaller and show branching defects. Scale bar 200μm. (**B**) Quantitative RT-PCR analysis showing ΔNp63 mRNA expression levels in salivary glands from control and ΔNp63cKO mice. (**C**) Immunofluorescence staining of E18.5 control and ΔNp63cKO salivary glands confirm the loss of ΔNp63 as well as the expression levels of K5, K14 and Sma stem/progenitor cell populations. Alterations to the acinar cell differentiation program is also observed in the ΔNp63cKO glands as evident by reduced expression of Aqp5 and Mist1. While K7 expression was unchanged, K19 expression was reduced in the mutant ducts. Scale bar 37μm. Data are represented as means ± SD (n = 3). **p<0.01.

During SMG embryonic morphogenesis, ΔNp63 is expressed in the epithelial cells of the developing placode and initial duct where it has been shown to play critical roles in directing cell fate choices and differentiation programs. To delineate the function of this transcription factor in SMG morphogenesis, we generated inducible UBC^*CreERT2*^;ΔNp63^*fl/GFP*^ (ΔNp63cKO) mice by mating ΔNp63^*fl/GFP*^ mice which contain one floxed allele and one allele in which the coding sequence of the ΔNp63-specific exon 3’ has been replaced by GFP [23, 24], with ubiquitously expressed inducible CreERT2 (UBC^*CreERT2*^) mice. Importantly, this approached allowed for overcoming the perinatal lethality associated with the loss of ΔNp63 [21, 23, 24]. Tamoxifen (TAM) was administered to pregnant females at embryonic (E) day 12.5 (E12.5), and embryonic glands were examined at E18.5. Histological analysis of hematoxylin and eosin (H&E) stained SMGs from ΔNp63cKO mice appeared smaller as compared to the ΔNp63^fl/fl^ (control) mice with obvious defects in branching morphogenesis (Fig 1A). Loss of ΔNp63 expression was confirmed at the mRNA level by quantitative reverse transcription polymerase chain reaction (qRT-PCR) analysis (Fig 1B).

To better evaluate the cellular alterations resulting from the loss of ΔNp63, we performed immunofluorescence analysis and co-stained the glands with ΔNp63 and the basal/myoepithelial marker, keratin 14 (K14). As expected, we observed a dramatic loss of ΔNp63 protein expression in the ΔNp63cKO glands compared to control glands, further confirming the loss of ΔNp63 (Fig 1C). Similarly, we detected a significant decrease in K14 expression in the ΔNp63cKO glands (Fig 1C). Examination of additional basal and myoepithelial markers, including K5 and smooth muscle actin (Sma), revealed dramatic differences in the KO glands when compared to the control, suggesting a loss of these progenitor cell populations (Fig 1C). While assessment of the ductal markers showed no appreciable difference in K8 or K7 expression in the KO glands, K19 expression was dramatically decreased in the ΔNp63cKO glands when compared to the control (Fig 1C). Indeed, the observed loss of both K5 and K19 expression in the KO glands is in good agreement with a previous study demonstrating that K19^+^ ductal cells are derived from K5^+^ progenitor cells [25]. Interestingly, evaluation of the acinar markers Aqp5 and Mist1 in both control and mutant SMGs showed altered morphology and reduced numbers of Mist1^+^ cells in the ΔNp63cKO glands, suggesting alterations to the acinar secretory cells (Fig 1C). Overall, our observations suggest that ablation of ΔNp63 during early gland morphogenesis results in alterations to the acinar secretory cells and ductal cell differentiation program, further highlighting the importance of ΔNp63 in regulating reciprocal signaling pathways during branching morphogenesis.

### Reduced cell proliferation in the SMGs of ΔNp63cKO mice

**Fig 2:**
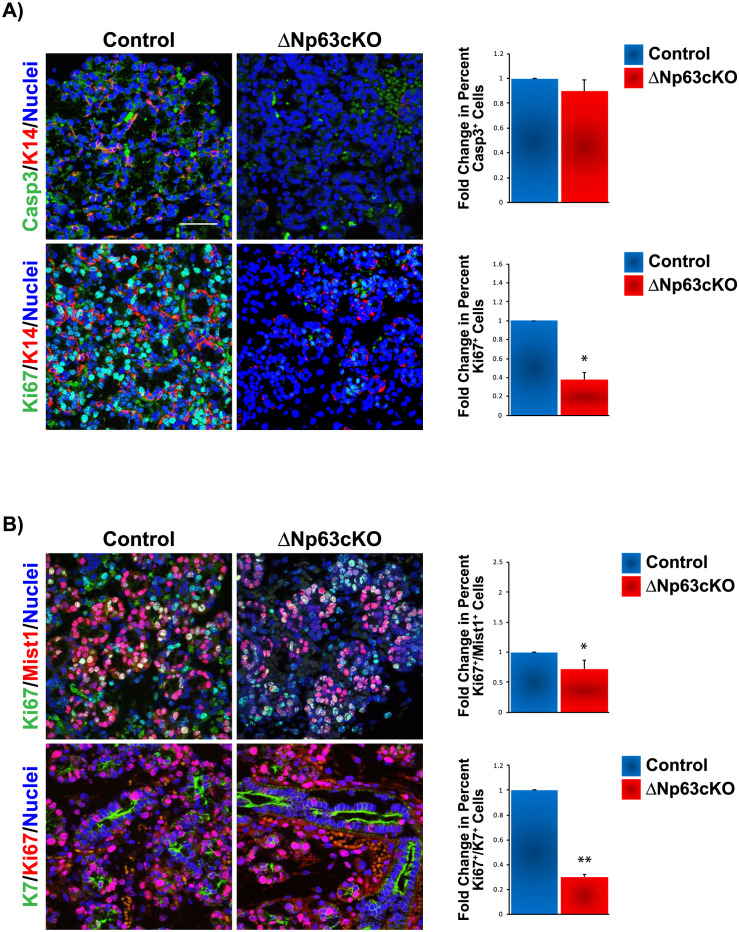
Reduced cell proliferation in ΔNp63cKO glands mice. (**A**) Expression and quantification analysis of apoptosis based on cleaved caspase-3 (Casp3) expression shows no differences between control and KO glands. Evaluation of cell proliferation based on Ki67 expression reveals decreased proliferation in the ΔNp63cKO glands compared to the control. (**B**) Expression and quantification analysis of Ki67 reveals reduced proliferation in both the acinar and ductal cells of the mutant glands compared to control. Scale bar 37μm. Data are represented as means ± SD (n = 3). *p<0.05, **p<0.01.

In light of the observed branching defects seen in the SMGs of the ΔNp63cKO mice together with the significant alterations to the ductal cell differentiation program, we wondered whether these changes could be a consequence of increased cell apoptosis. Although the KO glands revealed no significance changes in apoptosis, based on the expression of the apoptotic marker cleaved caspase-3 (Casp3), we did observe a moderate decrease in proliferation based on the proliferation marker Ki67, as compared to the control glands (Fig 2A). Given the decreased levels of proliferation in the KO glands, we next sought to determine if there were differences in proliferation between the acinar and duct cell populations. Towards this end, glands were co-stained with Ki67 and Mist1 and Ki67 and K7 to assess the status of proliferation in the acinar and ductal cells, respectively. Interestingly, while we found a moderate reduction in the number of Ki67^+^ acinar cells in the KO glands compared to the controls, there was a significant reduction in the proliferation status in the ducts of the KO (Fig 2B). Taken together, these results suggest the branching defect observed in the SMGs of the ΔNp63cKO may be due to impaired differentiation of K5^+^ progenitor cells into ductal cells, subsequently resulting in reduced ductal cell proliferation.

### ΔNp63 drives branching morphogenesis by directly regulating *Sfrp1* expression

**Fig 3:**
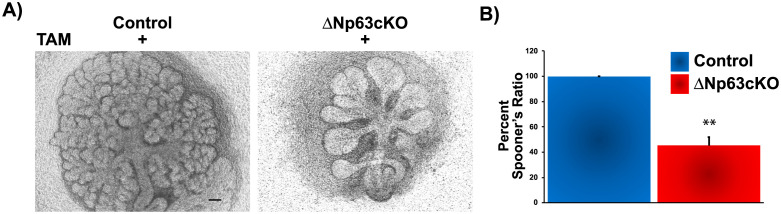
Branching morphogenesis of embryonic E13.5 SMGs from ΔNp63cKO mice is reduced in *ex vivo* culture. (**A**) Light micrographs of SMGs isolated from E13.5 control and ΔNp63cKO embryos and cultured *ex vivo* for 72 hours in the presence of Tamoxifen (TAM). (**B**) Spooner ratio quantification of the number of endbuds (expressed as a ratio of the number at 72h/the number at 1h) of control and KO SMGs. Scale bar 100μm. Data are represented as means ± SD (n = 3). **p<0.01.

To better evaluate the mechanisms through which ΔNp63 drives SG branching morphogenesis, we utilized an established *ex vivo* organ explant culture model system. Towards this end, E13.5 salivary gland rudiments from control and ΔNp63cKO embryos were harvested and cultured *ex vivo*. In order to knockdown (KD) ΔNp63 expression, media was supplemented with activated tamoxifen (TAM) and the glands were grown for 72 hours. Strikingly, genetic deletion of ΔNp63 resulted in a prominent reduction in overall branching which was accompanied by a significant decrease in the number of end buds (Fig 3A and 3B). These findings were further supported by the quantification of Spooner ratios, which showed a significant reduction in the number of end buds in KO glands compared to the control (Fig 3B). Moreover, the end buds of the KO glands appeared enlarged with a dramatic loss in the number of new clefts, compared to the control (Fig 3A).

**Fig 4:**
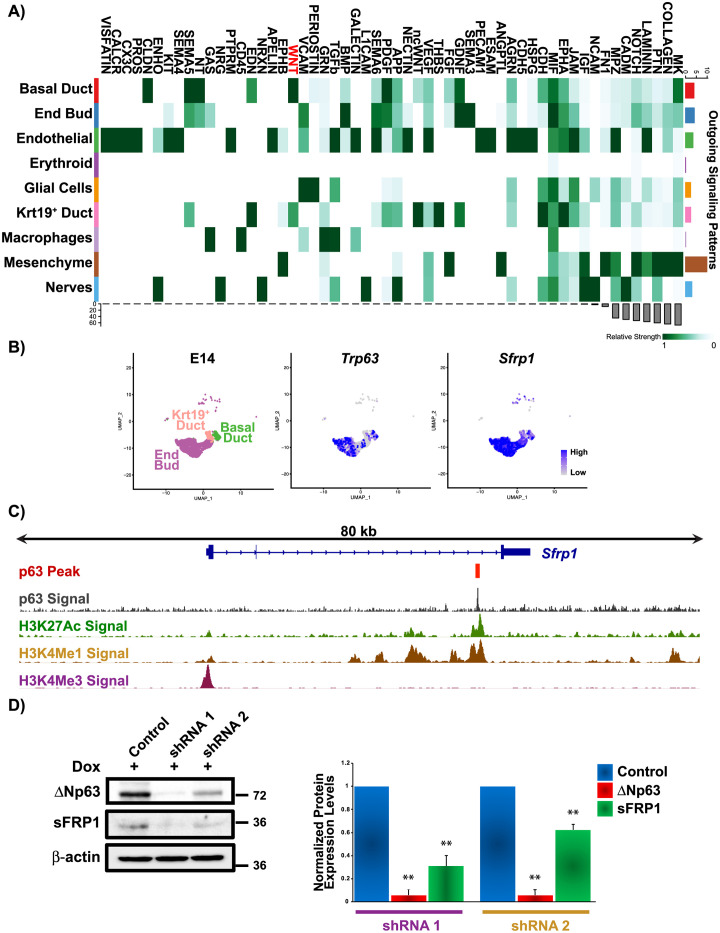
Direct regulation of Sfrp1 expression by ΔNp63. (**A**) Heatmap showing the summary of the signaling pathways that contribute to outgoing communication based on scRNA-seq [26]. The color bar represents the relative signaling strength of a signaling pathway across cell types. The grey bars indicate the sum of the signaling strength of each cell type or pathway. (**B**) Uniform manifold approximation and projection (UMAP) of E14 mouse SMG [26]. Epithelial cell populations are depicted. Feature plots demonstrating the expression of *Trp63* and *Sfrp1*. (**C**) Visualization of the ΔNp63 binding site identified within the *Sfrp1* genomic locus (Integrated Genomics Viewer). Top two lines display p63 ChIP binding site and signal enrichment in primary salivary gland epithelial cells(21). Overlays of histone ChIP-seq marking enhancers (H3K27ac and H3Kme1) and promoters (H3Kme3) are shown [21]. (**D**) Representative western blot analysis of mSG cells treated Dox which shows reduced protein expression levels of ΔNp63 and sFRP1 in the shRNA1 and shRNA2 infected knockdown cells as compared to the control cells. Densitometric analysis of the western blot is shown in the right panel. ΔNp63 and sFRP1 protein expression was normalized to β-actin. Data are represented as means ± SD (n = 3). **p<0.01.

To identify potential ΔNp63-driven signaling pathways that direct branching morphogenesis, we probed published single-cell RNA-seq (scRNA-seq) data sets from E14 SGs (GSE150327) [26]. Re-analysis of these data sets revealed 9 different cell populations, similar to what has been reported [26] (S1 Fig). To investigate the potential signaling communication patterns between the various cellular populations, we utilized CellChat [27]. Among the 9 cell types, several incoming and outgoing signaling pathways were predicted, many of which have been previously shown to play important roles in SG branching morphogenesis including BMP, FGF, Notch, laminin and collagen [28–30] (Fig 4A and S2 Fig). Considering the restricted expression pattern of ΔNp63 to the epithelial cells (Fig 4B), we focused on the three epithelial cell populations (End buds, Basal ducts and K19^+^ ducts) in order to identify p63-driven signaling pathways that may underlie the branching defects observed in the KO glands. Interestingly, evaluation of outgoing and incoming signaling patterns of the epithelial cells identified Wnt as a shared signaling pathway (Fig 4A and S2 Fig). Based on these findings and previous studies describing the role of Wnt signaling in branching morphogenesis, we further mined the scRNA-seq data sets to identify players in the Wnt signaling pathway fulfilling two criteria: 1) they share a similar expression pattern to that of ΔNp63 and 2) is a potential target gene of ΔNp63 based on our previous ChIP-seq studies [21]. Interestingly, our analysis identified the Wnt ligand antagonist, Secreted Frizzled Related Protein 1 (Sfrp1) [31], to be enriched in the epithelial cells of the SG at E14, similar to ΔNp63 (Fig 4B). Moreover, we identified a ΔNp63-bound region in exon 2 of the *Sfrp1* locus, suggesting that *Sfrp1* is a direct transcriptional target of ΔNp63 (Fig 4C). Armed with this information, we performed follow-up studies using a Doxycycline (Dox) inducible lentiviral-based delivery of shRNA to knockdown ΔNp63 expression in an immortalized mouse SMG salivary gland cell line (mSGc) [32]. ΔNp63 depletion was achieved using two independent shRNAs. Western blot analysis of mSGc treated with Dox revealed reduced protein expression levels of ΔNp63 and sFRP1 in the shRNA1 and shRNA2 infected knockdown cells as compared to the control cells (Fig 4D). Quantitative RT-PCR analysis of ΔNp63 and sFRP1 mRNAs in the ΔNp63 knockdown mSGc corroborated these findings (S3 Fig). Taken together these data strongly suggest that ΔNp63 can modulate Wnt signaling by directly regulating the expression of *Sfrp1*, which may account for the branching defects observed upon the loss of this transcription factor.

### The ΔNp63/Sfrp1 Wnt signaling axis regulates salivary gland branching

**Fig 5:**
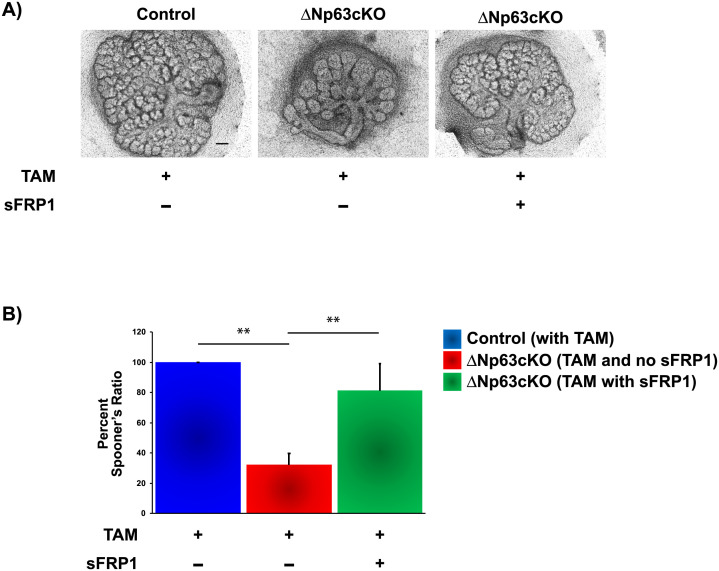
sFRP1 can rescue the branching defects observed upon the loss of ΔNp63. (**A**) Light micrographs of SMGs isolated from E13.5 control and ΔNp63cKO embryos and cultured *ex vivo* for 72 hours in the presence of Tamoxifen (TAM) and sFRP1 as indicated. (**B**) Spooner ratio quantification of control and ΔNp63cKO SMGs as described in panel A. Scale bar 100μm. Data are represented as means ± SD (n = 3). **p<0.01.

To determine the effects of sFRP1 on branching, wild type E13.5 SGs were treated with recombinant sFRP1. Interestingly, addition of sFRP1 resulted in a significant increase in branching based on Spooner branch ratios when compared to control wild type glands, confirming prior studies [10] (S4 Fig). Having established the effects of sFRP1 on branching morphogenesis, we wondered if the addition of sFRP1 could rescue the branching defect observed upon the loss of ΔNp63. Towards this end, control and ΔNp63cKO glands were cultured *ex vivo* and treated with TAM in the presence or absence of sFRP1. Indeed, compared with TAM-treated ΔNp63cKO SG alone, ΔNp63cKO SGs treated with TAM and sFRP1 resulted in increased branching suggesting that sFRP1 is able to compensate for the loss of ΔNp63 expression and partially restore branching (Fig 5).

## Discussion

Salivary gland branching morphogenesis is a complex developmental process that requires the precise temporal and spatial expression of genes driven by a network of transcription factors, signaling and regulatory molecules that coordinate these vital processes. Although we and others have reported the indispensable role of ΔNp63 in salivary gland morphogenesis, mechanistic studies in the SMG have been limited due to a lack of suitable model systems. Here we utilized *in vivo* conditional knockout mouse model to delete ΔNp63 during embryonic SMG development together with *ex vivo* embryonic salivary gland explants to tease out the functional consequences of temporal loss of ΔNp63. We find that ablation of ΔNp63 during embryogenesis resulted in broad alterations including a loss of the stem/progenitor cell population, impaired ductal and acinar cell differentiation leading to an overall defect in branching morphogenesis.

While the molecular mechanisms underlying ΔNp63 function in development and differentiation of the stratified epithelium such as the skin has been extensively reported in the literature [33, 34], its role in branching programs in various organs has been relatively less studied, particularly in the embryonic context. Our findings reported here, however, do not come as a complete surprise and fits well with the broad role of ΔNp63 in other tissues. This includes for example, the importance of ΔNp63-positive stem/progenitor cells of the urogenital sinus to generate all epithelial lineages of the prostate and bladder and in the differentiation program of the ureteric collecting duct in renal branching morphogenesis [35, 36]. Interestingly, these molecular attributes of ΔNp63 are likely to also be relevant in adult tissues, as evident from our previous studies in the SMG of adult ΔNp63cKO animals, where loss of ΔNp63 leads to aberrant ductal differentiation and smaller ducts as compared to control glands [21]. Similarly, conditional deletion of ΔNp63 in pubertal and lactating mammary glands of mice show broad ranging alterations in ductal formation and luminal cell differentiation [37, 38]. Taken together, these results firmly establish ΔNp63 as a key transcriptional regulator of branched and tubular structures, both during embryogenesis and in adult organs.

Our finding that *Sfrp1* is a direct p63 target gene that acts as an antagonist of the Wnt signaling pathway in the developing salivary gland raises the tantalizing possibility that this mechanism might act as an on-off switch. Presumably, as the branching process reaches the end state, the p63-*Sfrp1* axis is then counteracted by yet unknown processes which unencumber the developing salivary gland from the inhibited Wnt signaling state. One possibility is the potential epigenetic silencing of sFRP1 as observed in cancer cells [39]. Another interesting aspect of our findings dovetails well with the established and emerging role of Fgf signaling in the developing SMG [10, 40–42]. Indeed, recent studies have shown that loss of Fgf signaling leads to defects in salivary gland branching that is anchored by defective cell-cell and cell-matrix adhesion [43]. The fact that these same cellular processes are also under the purview of p63’s broad transcriptional control, suggests the strong possibility of signaling crosstalk between Wnt and Fgf signaling. This remains an exciting area for future research that can be pursued using both genetic knockout and salivary gland explant models described in this study.

One underdeveloped aspect of our investigation is that we examine the effects of loss of ΔNp63 during late embryogenesis, at which time myriad cell types that constitute the SMG have already been established. This choice is somewhat unavoidable given the limitations associated with the conditional KO model. For instance, our unpublished observations from TAM administration at an earlier time point, shows a major block in early SMG gland development, often resulting in complete agenesis of this organ. This precludes *in vivo* stepwise studies of the SMG at, for example the E14.5-E16.5 developmental window. Another challenge that is worth mentioning is the lack of a complete knockout in the ΔNp63 conditional KO model—indeed, we see a modest level of residual and persistent ΔNp63 that must be considered while interpreting our results. In the same vein, we show that while sFRP1 is able to compensate for the loss of ΔNp63 expression in the SMG explant system, branching is only partially restored. This suggests that additional ΔNp63-dependent signaling pathways are likely at play—our future studies will focus on some of these unresolved questions. Additionally, given that adult KO of ΔNp63 leads to a loss of Amy1 expression, reduced Aqp5 expression, and reduced saliva generation, it will be interesting to decipher the cell non-autonomous effects of ΔNp63 that extend to acinar cell lineage commitment and cell differentiation during embryogenesis.

## Material and methods

### Ethic approval

All animal experiments and procedures were performed in accordance with the State University of New York at Buffalo (University at Buffalo) Institutional Animal Care and Use Committee (IACUC) regulations. All procedures were approved by University at Buffalo IACUC (Protocol number: ORB10074Y). Pregnant females were euthanized by CO2 inhalation followed by cervical dislocation, which is the standard recommended method. Embryos were removed and euthanized via decapitation.

### Animal experiments

C57BL/6J (Stock No. 000664) and UBC^CreERT2^ (B6.Cg-Ndor1^Tg(UBC-cre/ERT2)1Ejb^/1J; Stock No. 007001) mice were purchased from The Jackson Laboratory (Bar Harbor, Maine). The ΔNp63-floxed (ΔNp63^fl/fl^) mice were provided by Elsa Flores and have been described previously [23]. The ΔNp63-GFP (ΔNp63^+/gfp^) mice were generated in our lab and have been previously described [24]. All mice were maintained on a C57BL/6J background. For timed pregnancies, mice were mated and noon of the day the vaginal plug was observed was considered E0.5. To induce Cre-LoxP recombination for the *in vivo* knockout studies, 2 mg of the inactive form of tamoxifen (TAM) (Sigma) dissolved in corn oil was administered to the embryos by intraperitoneal (IP) injections to pregnant females for three consecutive days (E12.5–14.5). Embryos were harvested at E18.5. Mice were euthanized by CO_2_ inhalation at E18.5 and the submandibular glands were harvested from the embryo through decapitation. For the *ex vivo* explant studies, the submandibular/sublingual gland from E13.5 control and *UBC*^*CreERT2*^;*ΔNp63*^*fl/gfp*^ embryo were dissected and cultured on 12 mm Transwell^®^ with 3.0 μm Pore Polycarbonate Membrane Insert (Corning) at the air/liquid interface floating on *ex vivo* culture medium in 12 well plates as described by Steinberg et al. [44]. Salivary gland rudiments were cultured on the membrane floating on *ex vivo* culture medium containing DMEM/F12 supplemented with 100U/ml penicillin, 100μg/ml streptomycin, 150μg/ml ascorbic acid, and 50μg/ml transferrin. For conditional deletion, salivary glands were cultured for 72 hours with 2μM of activated tamoxifen (4-OHT) supplemented culture medium. For the rescue experiments, salivary glands were cultured for 72 hours in the presence of 250ng/ml recombinant sFRP1 (R&D Systems). All glands were cultured at 37°C in 5% CO_2_ and the culture medium was changed every 24 hours.

### Immunostaining and imaging

Paraffin embedded submandibular gland tissue sections were processed for immunofluorescence analysis as previously described [22]. Primary antibodies used at the indicated dilutions include ΔNp63 (1:50, Cell Signaling Technology, D2K8X), K5 (1:100, gift from Dr. Julie Segre), K14 (1:100 [45]), alpha-smooth muscle actin (Sma) (1:200, Sigma, 1A4), Aqp5 (1:100, Alomone Labs), K7 (1:50, Abcam), Mist1 (1:100, Abcam), Troma-III (K19, 1:50, Development Studies Hybridoma Bank), Ki67 (1:100, Leica Biosystems, MM1), and Cleaved Caspase-3 (1:100, Cell Signaling Technology, D175). Sections were stained with TOPRO (Invitrogen) and mounted using VECTASHIELD Antifade Mounting Medium (Vector Laboratories) and imaged using an Andor Dragonfly Spinning Disk Confocal Microscope with Fiji [46]. Microscopy data in this study was acquired at the Optical Imaging and Analysis Facility, School of Dental Medicine, State University of New York at Buffalo.

### Quantification of cell proliferation and cell apoptosis

All immunostaining quantification analyses were performed using 400x confocal images and quantified using Image J (NIH; Bethesda, Maryland). A total of 10 fields of view (400x) were used for each quantification analysis described below. Cellular apoptosis was calculated by quantifying the number of Casp3^+^ cells which was divided by the total cell number. Approximately 350±50 cells were counted per field and a total of ~4,000±300 cells were counted per animal (n = 3). Cell proliferation was calculated by quantifying the number of Ki67^+^ cells which was divided by the total cell number. Approximately 350±50 cells were counted per field and a total of ~4,000±250 cells were counted per animal (n = 3). The percentage of Ki67^+^ cells that co-express Mist1 or K7 were calculated by counting the number of double positive Ki67^+^ and Mist1^+^ or K7^+^ cells, divided by the total number of Ki67^+^ cells (n = 3). Quantified values were reported as mean ± standard deviation (S.D.) of three or more independent experiments.

### Spooner’s ratio quantification

The number of end buds were counted at the time of dissection (E13.5) and at the end of the experiment (72hrs) using ImageJ. Spooner ratios were calculated by dividing the final endbud number by the initial end bud number for each explant [47]. Paired *t*-test was used to determine significance. Quantified values were reported as mean ± standard deviation (S.D.) of three or more independent experiments.

### Quantification and statistical analysis

Quantified results were reported as mean ± standard deviation (S.D.) of at least three or more independent experiments. Data comparison between the control and knockout samples were performed with two-tailed Student’s t-test with false discovery rate of less than 5%.

### Generation of p63 knockdown of Mouse Salivary Gland Epithelial Cells (mSGs)

The p63 shRNAs were generated using the pLKO-Tet-On lentivirus system. Briefly, 3 different regions of the mouse *Trp63* gene were chosen (S1 Table) for cloning into the pLKO-Tet-On plasmid using the Age1/EcoR1 restriction sites. The lentivirus was generated at the gene modulation core facility at the Roswell Park Comprehensive Cancer Center, Buffalo, NY. Mouse salivary gland epithelial cells (mSGc) [32] were transduced with the virus in the presence of 4 μg/ml Polybrene to aid transduction efficiency. Stably transduced target cells were selected using 2 μg/ml puromycin. Cells were treated with 2 μg/ml of doxycycline to knockdown p63 expression.

### Protein extraction and western blot analysis

Control and p63 depleted mSGc were harvested and proteins were extracted from cultured cells in RIPA buffer containing 2μg/ml phosphatase and protease inhibitor cocktail. Antibodies were diluted in 5% milk in TBST. Primary antibodies used were p63α (1:10,000, Cell Signaling Technology, D2K8X), sFRP1 (1:7,500, Invitrogen, JA11-68), β-actin (1:10,000, Cell Signaling Technology, 13E5). The blot was stripped using stripping buffer (Thermo Fisher) and was re-probed with β-actin for normalization (n = 3).

### RNA isolation and real-time quantitative reverse transcription PCR (qRT-PCR)

Total RNA was extracted by resuspending the mSGc in Trizol reagent (Thermo Fisher Scientific) using BioMashers (TaKaRa). The RNA was phase separated by chloroform and further isolated using the Direct-zol RNA Miniprep kit (Zymo Research). Isolated RNA was reverse transcribed using the iScript cDNA Synthesis kit (Bio-Rad) according to the manufacturer’s instructions and qRT-PCR was performed on a CFX96 Touch Real-Time PCR Detection System (Bio-Rad) using iQ SYBR Green Supermix (Bio-Rad). All qRT-PCR assays were performed in triplicates in at least three independent experiments. Relative expression values of each target gene were normalized to hypoxanthine guanine phosphoribosyltransferase (*Hprt*) expression. See S1 Table for primers sequences.

### Chromatin Immunoprecipitation-sequencing (ChIP-seq) and single cell RNA-sequencing (scRNA-seq) data sets

Previously published ChIP-seq datasets (GSE145264) was mapped to the *Mus musculus* genome (mm9 build) and ChIP-seq signals were visualized by using Integrative Genomics Viewer (IGV) [21, 48]. Additionally, scRNA-seq datasets were used to analyze the *Trp63* and *Sfrp1* expression pattern in the salivary gland at E14 (GSE150327) [26].

## Supporting information

S1 Fig Uniform manifold approximation and projection (UMAP) of E14 mouse SMG(26). Cell cluster identities are also shown.
(EPS)

S2 Fig Heatmap showing the summary of the signaling pathways that contribute to incoming communication based on scRNA-seq(26). The color bar represents the relative signaling strength of a signaling pathway across cell types. The bars indicate the sum of the signaling strength of each cell type or pathway.
(EPS)

S3 Fig Quantitative RT-PCR analysis showing *ΔNp63* and *Sfrp1* mRNA expression levels in mSG cells treated Dox which demonstrates reduced expression levels of *ΔNp63* and *Sfrp1* in the shRNA1 and shRNA2 infected knockdown cells as compared to the control cells. Values were normalized to the housekeeping gene *Hprt*. Data are represented as means ± SD (n = 3). **p<0.01.
(EPS)

S4 Fig (**A**) Light micrographs of SMGs isolated from wild type embryos and cultured *ex vivo* for 72 hours in the presence of sFRP1, as indicated. (**B**) Spooner ratio quantification. Data are represented as means ± SD (n = 3). **p<0.01.
(EPS)

S1 Table List of primers. (PDF)

S1 Raw images Original western blot image in Fig 4D. (EPS)
