# Supplementary material for: ΔNp63 regulates Sfrp1 expression to direct salivary gland branching morphogenesis
Source: PLoS One. 2024 May 9;19(5):e0301082. doi: 10.1371/journal.pone.0301082 (PMC11081224; doi:10.1371/journal.pone.0301082)
Supplement: S1 Table — (PDF) [file pone.0301082.s005.pdf]

S1 Table.

Gene	Forward	Reverse
∆Np63	5’-TGC CCA GAC TCA ATT TAG TGA GC-3’	5’-GAC GAG GAG CCG TTC TGA ATC-3’
Sfrp1	5’-CCA ACA GTG GGA ATT GAG GTA AG-3’	5’-CAC AGCAGA GAC TAT CCC TC-3’
Hprt	5’-CCT CAT GGA CTG ATT ATG GAC AG-3’	5’-TCA GCA AAG AAC TTA TAG CCC C-3’
shRNA#1	5’-AAT TAA AAA ACC GAG GTT GTG AAA CGA TGC CTC GAG GCA TCG TTT CAC AAC CTC GGT-3’	5’-CCG GAC CGA GGT TGT GAA ACG ATG CCT CGA GGC ATC GTT TCA CAA CCT CGG TTT TTT-3’
shRNA#2	5’-CCG GCT TCC TGT GTG TTG TAA GTA TCT CGA GAT ACT TAC AAC ACA CAG GAA GTT TTT-3’	5’-AAT TAA AAA CTT CCT GTG TGT TGT AAG TAT CTC GAG ATA CTT ACA ACA CAC AGG AAG-3’
